# Supplementary material for: Improving ascertainment of suicidal ideation and suicide attempt with natural language processing
Source: Sci Rep. 2022 Sep 7;12:15146. doi: 10.1038/s41598-022-19358-3 (PMC9452591; doi:10.1038/s41598-022-19358-3)
Supplement: Supplementary file 1 — Supplementary Information. [file 41598_2022_19358_MOESM1_ESM.pdf]

Improving ascertainment of suicidal ideation and suicide attempt with natural language processing

Supplemental Tables

Table S1 ICD9CM codes for suicidal ideation.

Table S2 ICD10CM codes for suicidal ideation.

Table S3 ICD9CM codes for suicide attempt.

Table S4 ICD10CM codes suicide attempt.

Table S5 Top 50 keywords generated by word2vec as semantically similar to 'suicide' and 'suicidal'.

Table S6 Query terms used to retrieve suicidal ideation and suicide attempt.

Table S7 Comparative analysis for extracting the top K highest ranked suicidal ideation and suicide attempt patients using various configurations of the suicide label assignment method.

Table S8 Suicidal ideation and suicide attempt cases extracted from the EHR.

Table S1 ICD9CM codes for suicidal ideation.

ICD9CM	Description
V62.84	Suicidal ideation

Table S2 ICD10CM codes for suicidal ideation.

ICD10CM	Description
R45.851	Suicidal ideations

Table S3 ICD9CM codes for suicide attempt.

ICD9CM	Description
E950*	Suicide and self-inflicted poisoning by solid or liquid substances
E951*	Suicide and self-inflicted poisoning by gases in domestic use
E952*	Suicide and self-inflicted poisoning by other gases and vapors
E953*	Suicide and self-inflicted injury by hanging, strangulation, and suffocation
E954	Suicide and self-inflicted injury by submersion [drowning]
E955*	Suicide and self-inflicted injury by firearms, air guns, and explosives
E956	Suicide and self-inflicted injury by cutting and piercing instrument
E957*	Suicide and self-inflicted injuries by jumping from high place
E958*	Suicide and self-inflicted injury by other and unspecified means
E959	Late effects of self-inflicted injury
E980*	Poisoning by solid or liquid substances, undetermined whether accidentally or purposely inflicted
E981*	Poisoning by gases in domestic use, undetermined whether accidentally or purposely inflicted
E982*	Poisoning by other gases, undetermined whether accidentally or purposely inflicted
E983*	Hanging, strangulation, or suffocation, undetermined whether accidentally or purposely inflicted
E984	Submersion (drowning), undetermined whether accidentally or purposely inflicted
E985*	Injury by firearms, air guns and explosives, undetermined whether accidentally or purposely inflicted
E986	Injury by cutting and piercing instruments, undetermined whether accidentally or purposely inflicted
E987*	Falling from high place, undetermined whether accidentally or purposely inflicted
E988*	Injury by other and unspecified means, undetermined whether accidentally or purposely inflicted
E989	Late effects of injury, undetermined whether accidentally or purposely inflicted

Table S4 ICD10CM codes suicide attempt.

ICD10CM	Description
T14.91*	Suicide attempt
T36.0X2*	Poisoning by penicillins, intentional self-harm
T36.0X4*	Poisoning by penicillins, undetermined
T36.1X2*	Poisoning by cephalosporins and other beta-lactam antibiotics, intentional self-harm
T36.1X4*	Poisoning by cephalosporins and other beta-lactam antibiotics, undetermined
T36.2X2*	Poisoning by chloramphenicol group, intentional self-harm
T36.2X4*	Poisoning by chloramphenicol group, undetermined
T36.3X2*	Poisoning by macrolides, intentional self-harm
T36.3X4*	Poisoning by macrolides, undetermined
T36.4X2*	Poisoning by tetracyclines, intentional self-harm
T36.4X4*	Poisoning by tetracyclines, undetermined
T36.5X2*	Poisoning by aminoglycosides, intentional self-harm
T36.5X4*	Poisoning by aminoglycosides, undetermined
T36.6X2*	Poisoning by rifampicins, intentional self-harm
T36.6X4*	Poisoning by rifampicins, undetermined

T36.7X2*	Poisoning by antifungal antibiotics, systemically used, intentional self-harm
T36.7X4*	Poisoning by antifungal antibiotics, systemically used, undetermined
T36.8X2*	Poisoning by other systemic antibiotics, intentional self-harm
T36.8X4*	Poisoning by other systemic antibiotics, undetermined
T36.92*	Poisoning by unspecified systemic antibiotic, intentional self-harm
T36.94*	Poisoning by unspecified systemic antibiotic, undetermined
T37.0X2*	Poisoning by sulfonamides, intentional self-harm
T37.0X4*	Poisoning by sulfonamides, undetermined
T37.1X2*	Poisoning by antimycobacterial drugs, intentional self-harm
T37.1X4*	Poisoning by antimycobacterial drugs, undetermined
T37.2X2*	Poisoning by antimalarials and drugs acting on other blood protozoa, intentional self-harm
T37.2X4*	Poisoning by antimalarials and drugs acting on other blood protozoa, undetermined
T37.3X2*	Poisoning by other antiprotozoal drugs, intentional self-harm
T37.3X4*	Poisoning by other antiprotozoal drugs, undetermined
T37.4X2*	Poisoning by anthelminthics, intentional self-harm
T37.4X4*	Poisoning by anthelminthics, undetermined
T37.5X2*	Poisoning by antiviral drugs, intentional self-harm
T37.5X4*	Poisoning by antiviral drugs, undetermined
T37.8X2*	Poisoning by other specified systemic anti-infectives and antiparasitics, intentional self-harm
T37.8X4*	Poisoning by other specified systemic anti-infectives and antiparasitics, undetermined
T37.92*	Poisoning by unspecified systemic anti-infective and antiparasitics, intentional self-harm
T37.94*	Poisoning by unspecified systemic anti-infective and antiparasitics, undetermined
T38.0X2*	Poisoning by glucocorticoids and synthetic analogues, intentional self-harm
T38.0X4*	Poisoning by glucocorticoids and synthetic analogues, undetermined
T38.1X2*	Poisoning by thyroid hormones and substitutes, intentional self-harm
T38.1X4*	Poisoning by thyroid hormones and substitutes, undetermined
T38.2X2*	Poisoning by antithyroid drugs, intentional self-harm
T38.2X4*	Poisoning by antithyroid drugs, undetermined
T38.3X2*	Poisoning by insulin and oral hypoglycemic [antidiabetic] drugs, intentional self-harm
T38.3X4*	Poisoning by insulin and oral hypoglycemic [antidiabetic] drugs, undetermined
T38.4X2*	Poisoning by oral contraceptives, intentional self-harm
T38.4X4*	Poisoning by oral contraceptives, undetermined
T38.5X2*	Poisoning by other estrogens and progestogens, intentional self-harm
T38.5X4*	Poisoning by other estrogens and progestogens, undetermined
T38.6X2*	Poisoning by antigonadotrophins, antiestrogens, antiandrogens, not elsewhere classified, intentional self-harm
T38.6X4*	Poisoning by antigonadotrophins, antiestrogens, antiandrogens, not elsewhere classified, undetermined
T38.7X2*	Poisoning by androgens and anabolic congeners, intentional self-harm
T38.7X4*	Poisoning by androgens and anabolic congeners, undetermined
T38.802*	Poisoning by unspecified hormones and synthetic substitutes, intentional self-harm
T38.804*	Poisoning by unspecified hormones and synthetic substitutes, undetermined
T38.812*	Poisoning by anterior pituitary [adenohypophyseal] hormones, intentional self-harm
T38.814*	Poisoning by anterior pituitary [adenohypophyseal] hormones, undetermined
T38.892*	Poisoning by other hormones and synthetic substitutes, intentional self-harm
T38.894*	Poisoning by other hormones and synthetic substitutes, undetermined
T38.902*	Poisoning by unspecified hormone antagonists, intentional self-harm
T38.904*	Poisoning by unspecified hormone antagonists, undetermined
T38.992*	Poisoning by other hormone antagonists, intentional self-harm
T38.994*	Poisoning by other hormone antagonists, undetermined
T39.012*	Poisoning by aspirin, intentional self-harm
T39.014*	Poisoning by aspirin, undetermined
T39.092*	Poisoning by salicylates, intentional self-harm

T39.094*	Poisoning by salicylates, undetermined
T39.1X2*	Poisoning by 4-Aminophenol derivatives, intentional self-harm
T39.1X4*	Poisoning by 4-Aminophenol derivatives, undetermined
T39.2X2*	Poisoning by pyrazolone derivatives, intentional self-harm
T39.2X4*	Poisoning by pyrazolone derivatives, undetermined
T39.312*	Poisoning by propionic acid derivatives, intentional self-harm
T39.314*	Poisoning by propionic acid derivatives, undetermined
T39.392*	Poisoning by other nonsteroidal anti-inflammatory drugs [NSAID], intentional self-harm
T39.394*	Poisoning by other nonsteroidal anti-inflammatory drugs [NSAID], undetermined
T39.4X2*	Poisoning by antirheumatics, not elsewhere classified, intentional self-harm
T39.4X4*	Poisoning by antirheumatics, not elsewhere classified, undetermined
T39.8X2*	Poisoning by other nonopioid analgesics and antipyretics, not elsewhere classified, intentional self-harm
T39.8X4*	Poisoning by other nonopioid analgesics and antipyretics, not elsewhere classified, undetermined
T39.92*	Poisoning by unspecified nonopioid analgesic, antipyretic and antirheumatic, intentional self-harm
T39.94*	Poisoning by unspecified nonopioid analgesic, antipyretic and antirheumatic, undetermined
T40.0X2*	Poisoning by opium, intentional self-harm
T40.0X4*	Poisoning by opium, undetermined
T40.1X2*	Poisoning by heroin, intentional self-harm
T40.1X4*	Poisoning by heroin, undetermined
T40.2X2*	Poisoning by other opioids, intentional self-harm
T40.2X4*	Poisoning by other opioids, undetermined
T40.3X2*	Poisoning by methadone, intentional self-harm
T40.3X4*	Poisoning by methadone, undetermined
T40.4X2*	Poisoning by other synthetic narcotics, intentional self-harm
T40.4X4*	Poisoning by other synthetic narcotics, undetermined
T40.5X2*	Poisoning by cocaine, intentional self-harm
T40.5X4*	Poisoning by cocaine, undetermined
T40.602*	Poisoning by unspecified narcotics, intentional self-harm
T40.604*	Poisoning by unspecified narcotics, undetermined
T40.692*	Poisoning by other narcotics, intentional self-harm
T40.694*	Poisoning by other narcotics, undetermined
T40.7X2*	Poisoning by cannabis (derivatives), intentional self-harm
T40.7X4*	Poisoning by cannabis (derivatives), undetermined
T40.8X2*	Poisoning by lysergide [LSD], intentional self-harm
T40.8X4*	Poisoning by lysergide [LSD], undetermined
T40.902*	Poisoning by unspecified psychodysleptics [hallucinogens], intentional self-harm
T40.904*	Poisoning by unspecified psychodysleptics [hallucinogens], undetermined
T40.992*	Poisoning by other psychodysleptics [hallucinogens], intentional self-harm
T40.994*	Poisoning by other psychodysleptics [hallucinogens], undetermined
T41.0X2*	Poisoning by inhaled anesthetics, intentional self-harm
T41.0X4*	Poisoning by inhaled anesthetics, undetermined
T41.1X2*	Poisoning by intravenous anesthetics, intentional self-harm
T41.1X4*	Poisoning by intravenous anesthetics, undetermined
T41.202*	Poisoning by unspecified general anesthetics, intentional self-harm
T41.204*	Poisoning by unspecified general anesthetics, undetermined
T41.292*	Poisoning by other general anesthetics, intentional self-harm
T41.294*	Poisoning by other general anesthetics, undetermined
T41.3X2*	Poisoning by local anesthetics, intentional self-harm
T41.3X4*	Poisoning by local anesthetics, undetermined
T41.42*	Poisoning by unspecified anesthetic, intentional self-harm
T41.44*	Poisoning by unspecified anesthetic, undetermined
T41.5X2*	Poisoning by therapeutic gases, intentional self-harm

T41.5X4*	Poisoning by therapeutic gases, undetermined
T42.0X2*	Poisoning by hydantoin derivatives, intentional self-harm
T42.0X4*	Poisoning by hydantoin derivatives, undetermined
T42.1X2*	Poisoning by iminostilbenes, intentional self-harm
T42.1X4*	Poisoning by iminostilbenes, undetermined
T42.2X2*	Poisoning by succinimides and oxazolidinediones, intentional self-harm
T42.2X4*	Poisoning by succinimides and oxazolidinediones, undetermined
T42.3X2*	Poisoning by barbiturates, intentional self-harm
T42.3X4*	Poisoning by barbiturates, undetermined
T42.4X2*	Poisoning by benzodiazepines, intentional self-harm
T42.4X4*	Poisoning by benzodiazepines, undetermined
T42.5X2*	Poisoning by mixed antiepileptics, intentional self-harm
T42.5X4*	Poisoning by mixed antiepileptics, undetermined
T42.6X2*	Poisoning by other antiepileptic and sedative-hypnotic drugs, intentional self-harm
T42.6X4*	Poisoning by other antiepileptic and sedative-hypnotic drugs, undetermined
T42.72*	Poisoning by unspecified antiepileptic and sedative-hypnotic drugs, intentional self-harm
T42.74*	Poisoning by unspecified antiepileptic and sedative-hypnotic drugs, undetermined
T42.8X2*	Poisoning by antiparkinsonism drugs and other central muscle-tone depressants, intentional self-harm
T42.8X4*	Poisoning by antiparkinsonism drugs and other central muscle-tone depressants, undetermined
T43.012*	Poisoning by tricyclic antidepressants, intentional self-harm
T43.014*	Poisoning by tricyclic antidepressants, undetermined
T43.022*	Poisoning by tetracyclic antidepressants, intentional self-harm
T43.024*	Poisoning by tetracyclic antidepressants, undetermined
T43.0X2*	Poisoning by tricyclic and tetracyclic antidepressants, intentional self-harm
T43.0X4*	Poisoning by tricyclic and tetracyclic antidepressants, undetermined
T43.1X2*	Poisoning by monoamine-oxidase-inhibitor antidepressants, intentional self-harm
T43.1X4*	Poisoning by monoamine-oxidase-inhibitor antidepressants, undetermined
T43.202*	Poisoning by unspecified antidepressants, intentional self-harm
T43.204*	Poisoning by unspecified antidepressants, undetermined
T43.212*	Poisoning by selective serotonin and norepinephrine reuptake inhibitors, intentional self-harm
T43.214*	Poisoning by selective serotonin and norepinephrine reuptake inhibitors, undetermined
T43.222*	Poisoning by selective serotonin reuptake inhibitors, intentional self-harm
T43.224*	Poisoning by selective serotonin reuptake inhibitors, undetermined
T43.292*	Poisoning by other antidepressants, intentional self-harm
T43.294*	Poisoning by other antidepressants, undetermined
T43.3X2*	Poisoning by phenothiazine antipsychotics and neuroleptics, intentional self-harm
T43.3X4*	Poisoning by phenothiazine antipsychotics and neuroleptics, undetermined
T43.4X2*	Poisoning by butyrophenone and thiothixene neuroleptics, intentional self-harm
T43.4X4*	Poisoning by butyrophenone and thiothixene neuroleptics, undetermined
T43.502*	Poisoning by unspecified antipsychotics and neuroleptics, intentional self-harm
T43.504*	Poisoning by unspecified antipsychotics and neuroleptics, undetermined
T43.592*	Poisoning by other antipsychotics and neuroleptics, intentional self-harm
T43.594*	Poisoning by other antipsychotics and neuroleptics, undetermined
T43.602*	Poisoning by unspecified psychostimulants, intentional self-harm
T43.604*	Poisoning by unspecified psychostimulants, undetermined
T43.612*	Poisoning by caffeine, intentional self-harm
T43.614*	Poisoning by caffeine, undetermined
T43.622*	Poisoning by amphetamines, intentional self-harm
T43.624*	Poisoning by amphetamines, undetermined
T43.632*	Poisoning by methylphenidate, intentional self-harm
T43.634*	Poisoning by methylphenidate, undetermined
T43.642*	Poisoning by ecstasy, intentional self-harm

T43.644*	Poisoning by ecstasy, undetermined
T43.692*	Poisoning by other psychostimulants, intentional self-harm
T43.694*	Poisoning by other psychostimulants, undetermined
T43.6X2*	Poisoning by psychostimulants with abuse potential, intentional self-harm
T43.6X4*	Poisoning by psychostimulants with abuse potential, undetermined
T43.8X2*	Poisoning by other psychotropic drugs, intentional self-harm
T43.8X4*	Poisoning by other psychotropic drugs, undetermined
T43.92*	Poisoning by unspecified psychotropic drug, intentional self-harm
T43.94*	Poisoning by unspecified psychotropic drug, undetermined
T44.0X2*	Poisoning by anticholinesterase agents, intentional self-harm
T44.0X4*	Poisoning by anticholinesterase agents, undetermined
T44.1X2*	Poisoning by other parasympathomimetics [cholinergics], intentional self-harm
T44.1X4*	Poisoning by other parasympathomimetics [cholinergics], undetermined
T44.2X2*	Poisoning by ganglionic blocking drugs, intentional self-harm
T44.2X4*	Poisoning by ganglionic blocking drugs, undetermined
T44.3X2*	Poisoning by other parasympatholytics [anticholinergics and antimuscarinics] and spasmolytics, intentional self-harm
T44.3X4*	Poisoning by other parasympatholytics [anticholinergics and antimuscarinics] and spasmolytics, undetermined
T44.4X2*	Poisoning by predominantly alpha-adrenoreceptor agonists, intentional self-harm
T44.4X4*	Poisoning by predominantly alpha-adrenoreceptor agonists, undetermined
T44.5X2*	Poisoning by predominantly beta-adrenoreceptor agonists, intentional self-harm
T44.5X4*	Poisoning by predominantly beta-adrenoreceptor agonists, undetermined
T44.6X2*	Poisoning by alpha-adrenoreceptor antagonists, intentional self-harm
T44.6X4*	Poisoning by alpha-adrenoreceptor antagonists, undetermined
T44.7X2*	Poisoning by beta-adrenoreceptor antagonists, intentional self-harm
T44.7X4*	Poisoning by beta-adrenoreceptor antagonists, undetermined
T44.8X2*	Poisoning by centrally-acting and adrenergic-neuron-blocking agents, intentional self-harm
T44.8X4*	Poisoning by centrally-acting and adrenergic-neuron-blocking agents, undetermined
T44.902*	Poisoning by unspecified drugs primarily affecting the autonomic nervous system, intentional self-harm
T44.904*	Poisoning by unspecified drugs primarily affecting the autonomic nervous system, undetermined
T44.992*	Poisoning by other drug primarily affecting the autonomic nervous system, intentional self-harm
T44.994*	Poisoning by other drug primarily affecting the autonomic nervous system, undetermined
T45.0X2*	Poisoning by antiallergic and antiemetic drugs, intentional self-harm
T45.0X4*	Poisoning by antiallergic and antiemetic drugs, undetermined
T45.1X2*	Poisoning by antineoplastic and immunosuppressive drugs, intentional self-harm
T45.1X4*	Poisoning by antineoplastic and immunosuppressive drugs, undetermined
T45.2X2*	Poisoning by vitamins, intentional self-harm
T45.2X4*	Poisoning by vitamins, undetermined
T45.3X2*	Poisoning by enzymes, intentional self-harm
T45.3X4*	Poisoning by enzymes, undetermined
T45.4X2*	Poisoning by iron and its compounds, intentional self-harm
T45.4X4*	Poisoning by iron and its compounds, undetermined
T45.512*	Poisoning by anticoagulants, intentional self-harm
T45.514*	Poisoning by anticoagulants, undetermined
T45.522*	Poisoning by antithrombotic drugs, intentional self-harm
T45.524*	Poisoning by antithrombotic drugs, undetermined
T45.602*	Poisoning by unspecified fibrinolysis-affecting drugs, intentional self-harm
T45.604*	Poisoning by unspecified fibrinolysis-affecting drugs, undetermined
T45.612*	Poisoning by thrombolytic drug, intentional self-harm
T45.614*	Poisoning by thrombolytic drug, undetermined
T45.622*	Poisoning by hemostatic drug, intentional self-harm

T45.624*	Poisoning by hemostatic drug, undetermined
T45.692*	Poisoning by other fibrinolysis-affecting drugs, intentional self-harm
T45.694*	Poisoning by other fibrinolysis-affecting drugs, undetermined
T45.7X2*	Poisoning by anticoagulant antagonists, vitamin K and other coagulants, intentional self-harm
T45.7X4*	Poisoning by anticoagulant antagonists, vitamin K and other coagulants, undetermined
T45.8X2*	Poisoning by other primarily systemic and hematological agents, intentional self-harm
T45.8X4*	Poisoning by other primarily systemic and hematological agents, undetermined
T45.92*	Poisoning by unspecified primarily systemic and hematological agent, intentional self-harm
T45.94*	Poisoning by unspecified primarily systemic and hematological agent, undetermined
T46.0X2*	Poisoning by cardiac-stimulant glycosides and drugs of similar action, intentional self-harm
T46.0X4*	Poisoning by cardiac-stimulant glycosides and drugs of similar action, undetermined
T46.1X2*	Poisoning by calcium-channel blockers, intentional self-harm
T46.1X4*	Poisoning by calcium-channel blockers, undetermined
T46.2X2*	Poisoning by other antidysrhythmic drugs, intentional self-harm
T46.2X4*	Poisoning by other antidysrhythmic drugs, undetermined
T46.3X2*	Poisoning by coronary vasodilators, intentional self-harm
T46.3X4*	Poisoning by coronary vasodilators, undetermined
T46.4X2*	Poisoning by angiotensin-converting-enzyme inhibitors, intentional self-harm
T46.4X4*	Poisoning by angiotensin-converting-enzyme inhibitors, undetermined
T46.5X2*	Poisoning by other antihypertensive drugs, intentional self-harm
T46.5X4*	Poisoning by other antihypertensive drugs, undetermined
T46.6X2*	Poisoning by antihyperlipidemic and antiarteriosclerotic drugs, intentional self-harm
T46.6X4*	Poisoning by antihyperlipidemic and antiarteriosclerotic drugs, undetermined
T46.7X2*	Poisoning by peripheral vasodilators, intentional self-harm
T46.7X4*	Poisoning by peripheral vasodilators, undetermined
T46.8X2*	Poisoning by antivaricose drugs, including sclerosing agents, intentional self-harm
T46.8X4*	Poisoning by antivaricose drugs, including sclerosing agents, undetermined
T46.902*	Poisoning by unspecified agents primarily affecting the cardiovascular system, intentional self-harm
T46.904*	Poisoning by unspecified agents primarily affecting the cardiovascular system, undetermined
T46.992*	Poisoning by other agents primarily affecting the cardiovascular system, intentional self-harm
T46.994*	Poisoning by other agents primarily affecting the cardiovascular system, undetermined
T47.0X2*	Poisoning by histamine H2-receptor blockers, intentional self-harm
T47.0X4*	Poisoning by histamine H2-receptor blockers, undetermined
T47.1X2*	Poisoning by other antacids and anti-gastric-secretion drugs, intentional self-harm
T47.1X4*	Poisoning by other antacids and anti-gastric-secretion drugs, undetermined
T47.2X2*	Poisoning by stimulant laxatives, intentional self-harm
T47.2X4*	Poisoning by stimulant laxatives, undetermined
T47.3X2*	Poisoning by saline and osmotic laxatives, intentional self-harm
T47.3X4*	Poisoning by saline and osmotic laxatives, undetermined
T47.4X2*	Poisoning by other laxatives, intentional self-harm
T47.4X4*	Poisoning by other laxatives, undetermined
T47.5X2*	Poisoning by digestants, intentional self-harm
T47.5X4*	Poisoning by digestants, undetermined
T47.6X2*	Poisoning by antidiarrheal drugs, intentional self-harm
T47.6X4*	Poisoning by antidiarrheal drugs, undetermined
T47.7X2*	Poisoning by emetics, intentional self-harm
T47.7X4*	Poisoning by emetics, undetermined
T47.8X2*	Poisoning by other agents primarily affecting gastrointestinal system, intentional self-harm
T47.8X4*	Poisoning by other agents primarily affecting gastrointestinal system, undetermined
T47.92*	Poisoning by unspecified agents primarily affecting the gastrointestinal system, intentional self-harm
T47.94*	Poisoning by unspecified agents primarily affecting the gastrointestinal system, undetermined
T48.0X2*	Poisoning by oxytocic drugs, intentional self-harm

T48.0X4*	Poisoning by oxytocic drugs, undetermined
T48.1X2*	Poisoning by skeletal muscle relaxants [neuromuscular blocking agents], intentional self-harm
T48.1X4*	Poisoning by skeletal muscle relaxants [neuromuscular blocking agents], undetermined
T48.202*	Poisoning by unspecified drugs acting on muscles, intentional self-harm
T48.204*	Poisoning by unspecified drugs acting on muscles, undetermined
T48.292*	Poisoning by other drugs acting on muscles, intentional self-harm
T48.294*	Poisoning by other drugs acting on muscles, undetermined
T48.3X2*	Poisoning by antitussives, intentional self-harm
T48.3X4*	Poisoning by antitussives, undetermined
T48.4X2*	Poisoning by expectorants, intentional self-harm
T48.4X4*	Poisoning by expectorants, undetermined
T48.5X2*	Poisoning by other anti-common-cold drugs, intentional self-harm
T48.5X4*	Poisoning by other anti-common-cold drugs, undetermined
T48.6X2*	Poisoning by antiasthmatics, intentional self-harm
T48.6X4*	Poisoning by antiasthmatics, undetermined
T48.902*	Poisoning by unspecified agents primarily acting on the respiratory system, intentional self-harm
T48.904*	Poisoning by unspecified agents primarily acting on the respiratory system, undetermined
T48.992*	Poisoning by other agents primarily acting on the respiratory system, intentional self-harm
T48.994*	Poisoning by other agents primarily acting on the respiratory system, undetermined
T49.0X2*	Poisoning by local antifungal, anti-infective and anti-inflammatory drugs, intentional self-harm
T49.0X4*	Poisoning by local antifungal, anti-infective and anti-inflammatory drugs, undetermined
T49.1X2*	Poisoning by antipruritics, intentional self-harm
T49.1X4*	Poisoning by antipruritics, undetermined
T49.2X2*	Poisoning by local astringents and local detergents, intentional self-harm
T49.2X4*	Poisoning by local astringents and local detergents, undetermined
T49.3X2*	Poisoning by emollients, demulcents and protectants, intentional self-harm
T49.3X4*	Poisoning by emollients, demulcents and protectants, undetermined
T49.4X2*	Poisoning by keratolytics, keratoplastics, and other hair treatment drugs and preparations, intentional self-harm
T49.4X4*	Poisoning by keratolytics, keratoplastics, and other hair treatment drugs and preparations, undetermined
T49.5X2*	Poisoning by ophthalmological drugs and preparations, intentional self-harm
T49.5X4*	Poisoning by ophthalmological drugs and preparations, undetermined
T49.6X2*	Poisoning by otorhinolaryngological drugs and preparations, intentional self-harm
T49.6X4*	Poisoning by otorhinolaryngological drugs and preparations, undetermined
T49.7X2*	Poisoning by dental drugs, topically applied, intentional self-harm
T49.7X4*	Poisoning by dental drugs, topically applied, undetermined
T49.8X2*	Poisoning by other topical agents, intentional self-harm
T49.8X4*	Poisoning by other topical agents, undetermined
T49.92*	Poisoning by unspecified topical agent, intentional self-harm
T49.94*	Poisoning by unspecified topical agent, undetermined
T50.0X2*	Poisoning by mineralocorticoids and their antagonists, intentional self-harm
T50.0X4*	Poisoning by mineralocorticoids and their antagonists, undetermined
T50.1X2*	Poisoning by loop [high-ceiling] diuretics, intentional self-harm
T50.1X4*	Poisoning by loop [high-ceiling] diuretics, undetermined
T50.2X2*	Poisoning by carbonic-anhydrase inhibitors, benzothiadiazides and other diuretics, intentional self-harm
T50.2X4*	Poisoning by carbonic-anhydrase inhibitors, benzothiadiazides and other diuretics, undetermined
T50.3X2*	Poisoning by electrolytic, caloric and water-balance agents, intentional self-harm
T50.3X4*	Poisoning by electrolytic, caloric and water-balance agents, undetermined
T50.4X2*	Poisoning by drugs affecting uric acid metabolism, intentional self-harm
T50.4X4*	Poisoning by drugs affecting uric acid metabolism, undetermined
T50.5X2*	Poisoning by appetite depressants, intentional self-harm

T50.5X4*	Poisoning by appetite depressants, undetermined
T50.6X2*	Poisoning by antidotes and chelating agents, intentional self-harm
T50.6X4*	Poisoning by antidotes and chelating agents, undetermined
T50.7X2*	Poisoning by analeptics and opioid receptor antagonists, intentional self-harm
T50.7X4*	Poisoning by analeptics and opioid receptor antagonists, undetermined
T50.8X2*	Poisoning by diagnostic agents, intentional self-harm
T50.8X4*	Poisoning by diagnostic agents, undetermined
T50.902*	Poisoning by unspecified drugs, medicaments and biological substances, intentional self-harm
T50.904*	Poisoning by unspecified drugs, medicaments and biological substances, undetermined
T50.992*	Poisoning by other drugs, medicaments and biological substances, intentional self-harm
T50.994*	Poisoning by other drugs, medicaments and biological substances, undetermined
T50.A12*	Poisoning by pertussis vaccine, including combinations with a pertussis component, intentional self-harm
T50.A14*	Poisoning by pertussis vaccine, including combinations with a pertussis component, undetermined
T50.A22*	Poisoning by mixed bacterial vaccines without a pertussis component, intentional self-harm
T50.A24*	Poisoning by mixed bacterial vaccines without a pertussis component, undetermined
T50.A92*	Poisoning by other bacterial vaccines, intentional self-harm
T50.A94*	Poisoning by other bacterial vaccines, undetermined
T50.B12*	Poisoning by smallpox vaccines, intentional self-harm
T50.B14*	Poisoning by smallpox vaccines, undetermined
T50.B92*	Poisoning by other viral vaccines, intentional self-harm
T50.B94*	Poisoning by other viral vaccines, undetermined
T50.Z12*	Poisoning by immunoglobulin, intentional self-harm
T50.Z14*	Poisoning by immunoglobulin, undetermined
T50.Z92*	Poisoning by other vaccines and biological substances, intentional self-harm
T50.Z94*	Poisoning by other vaccines and biological substances, undetermined
T51.0X2*	Toxic effect of ethanol, intentional self-harm
T51.0X4*	Toxic effect of ethanol, undetermined
T51.1X2*	Toxic effect of methanol, intentional self-harm
T51.1X4*	Toxic effect of methanol, undetermined
T51.2X2*	Toxic effect of 2-Propanol, intentional self-harm
T51.2X4*	Toxic effect of 2-Propanol, undetermined
T51.3X2*	Toxic effect of fusel oil, intentional self-harm
T51.3X4*	Toxic effect of fusel oil, undetermined
T51.8X2*	Toxic effect of other alcohols, intentional self-harm
T51.8X4*	Toxic effect of other alcohols, undetermined
T51.92*	Toxic effect of unspecified alcohol, intentional self-harm
T51.94*	Toxic effect of unspecified alcohol, undetermined
T52.0X2*	Toxic effect of petroleum products, intentional self-harm
T52.0X4*	Toxic effect of petroleum products, undetermined
T52.1X2*	Toxic effect of benzene, intentional self-harm
T52.1X4*	Toxic effect of benzene, undetermined
T52.2X2*	Toxic effect of homologues of benzene, intentional self-harm
T52.2X4*	Toxic effect of homologues of benzene, undetermined
T52.3X2*	Toxic effect of glycols, intentional self-harm
T52.3X4*	Toxic effect of glycols, undetermined
T52.4X2*	Toxic effect of ketones, intentional self-harm
T52.4X4*	Toxic effect of ketones, undetermined
T52.8X2*	Toxic effect of other organic solvents, intentional self-harm
T52.8X4*	Toxic effect of other organic solvents, undetermined
T52.92*	Toxic effect of unspecified organic solvent, intentional self-harm
T52.94*	Toxic effect of unspecified organic solvent, undetermined
T53.0X2*	Toxic effect of carbon tetrachloride, intentional self-harm

T53.0X4*	Toxic effect of carbon tetrachloride, undetermined
T53.1X2*	Toxic effect of chloroform, intentional self-harm
T53.1X4*	Toxic effect of chloroform, undetermined
T53.2X2*	Toxic effect of trichloroethylene, intentional self-harm
T53.2X4*	Toxic effect of trichloroethylene, undetermined
T53.3X2*	Toxic effect of tetrachloroethylene, intentional self-harm
T53.3X4*	Toxic effect of tetrachloroethylene, undetermined
T53.4X2*	Toxic effect of dichloromethane, intentional self-harm
T53.4X4*	Toxic effect of dichloromethane, undetermined
T53.5X2*	Toxic effect of chlorofluorocarbons, intentional self-harm
T53.5X4*	Toxic effect of chlorofluorocarbons, undetermined
T53.6X2*	Toxic effect of other halogen derivatives of aliphatic hydrocarbons, intentional self-harm
T53.6X4*	Toxic effect of other halogen derivatives of aliphatic hydrocarbons, undetermined
T53.7X2*	Toxic effect of other halogen derivatives of aromatic hydrocarbons, intentional self-harm
T53.7X4*	Toxic effect of other halogen derivatives of aromatic hydrocarbons, undetermined
T53.92*	Toxic effect of unspecified halogen derivatives of aliphatic and aromatic hydrocarbons, intentional self-harm
T53.94*	Toxic effect of unspecified halogen derivatives of aliphatic and aromatic hydrocarbons, undetermined
T54.0X2*	Toxic effect of phenol and phenol homologues, intentional self-harm
T54.0X4*	Toxic effect of phenol and phenol homologues, undetermined
T54.1X2*	Toxic effect of other corrosive organic compounds, intentional self-harm
T54.1X4*	Toxic effect of other corrosive organic compounds, undetermined
T54.2X2*	Toxic effect of corrosive acids and acid-like substances, intentional self-harm
T54.2X4*	Toxic effect of corrosive acids and acid-like substances, undetermined
T54.3X2*	Toxic effect of corrosive alkalis and alkali-like substances, intentional self-harm
T54.3X4*	Toxic effect of corrosive alkalis and alkali-like substances, undetermined
T54.92*	Toxic effect of unspecified corrosive substance, intentional self-harm
T54.94*	Toxic effect of unspecified corrosive substance, undetermined
T55.0X2*	Toxic effect of soaps, intentional self-harm
T55.0X4*	Toxic effect of soaps, undetermined
T55.1X2*	Toxic effect of detergents, intentional self-harm
T55.1X4*	Toxic effect of detergents, undetermined
T56.0X2*	Toxic effect of lead and its compounds, intentional self-harm
T56.0X4*	Toxic effect of lead and its compounds, undetermined
T56.1X2*	Toxic effect of mercury and its compounds, intentional self-harm
T56.1X4*	Toxic effect of mercury and its compounds, undetermined
T56.2X2*	Toxic effect of chromium and its compounds, intentional self-harm
T56.2X4*	Toxic effect of chromium and its compounds, undetermined
T56.3X2*	Toxic effect of cadmium and its compounds, intentional self-harm
T56.3X4*	Toxic effect of cadmium and its compounds, undetermined
T56.4X2*	Toxic effect of copper and its compounds, intentional self-harm
T56.4X4*	Toxic effect of copper and its compounds, undetermined
T56.5X2*	Toxic effect of zinc and its compounds, intentional self-harm
T56.5X4*	Toxic effect of zinc and its compounds, undetermined
T56.6X2*	Toxic effect of tin and its compounds, intentional self-harm
T56.6X4*	Toxic effect of tin and its compounds, undetermined
T56.7X2*	Toxic effect of beryllium and its compounds, intentional self-harm
T56.7X4*	Toxic effect of beryllium and its compounds, undetermined
T56.812*	Toxic effect of thallium, intentional self-harm
T56.814*	Toxic effect of thallium, undetermined
T56.892*	Toxic effect of other metals, intentional self-harm
T56.894*	Toxic effect of other metals, undetermined

T56.8X2*	Toxic effect of other metals, intentional self-harm
T56.8X4*	Toxic effect of other metals, undetermined
T56.92*	Toxic effect of unspecified metal, intentional self-harm
T56.94*	Toxic effect of unspecified metal, undetermined
T57.0X2*	Toxic effect of arsenic and its compounds, intentional self-harm
T57.0X4*	Toxic effect of arsenic and its compounds, undetermined
T57.1X2*	Toxic effect of phosphorus and its compounds, intentional self-harm
T57.1X4*	Toxic effect of phosphorus and its compounds, undetermined
T57.2X2*	Toxic effect of manganese and its compounds, intentional self-harm
T57.2X4*	Toxic effect of manganese and its compounds, undetermined
T57.3X2*	Toxic effect of hydrogen cyanide, intentional self-harm
T57.3X4*	Toxic effect of hydrogen cyanide, undetermined
T57.8X2*	Toxic effect of other specified inorganic substances, intentional self-harm
T57.8X4*	Toxic effect of other specified inorganic substances, undetermined
T57.92*	Toxic effect of unspecified inorganic substance, intentional self-harm
T57.94*	Toxic effect of unspecified inorganic substance, undetermined
T58.02*	Toxic effect of carbon monoxide from motor vehicle exhaust, intentional self-harm
T58.04*	Toxic effect of carbon monoxide from motor vehicle exhaust, undetermined
T58.12*	Toxic effect of carbon monoxide from utility gas, intentional self-harm
T58.14*	Toxic effect of carbon monoxide from utility gas, undetermined
T58.2X2*	Toxic effect of carbon monoxide from incomplete combustion of other domestic fuels, intentional self-harm
T58.2X4*	Toxic effect of carbon monoxide from incomplete combustion of other domestic fuels, undetermined
T58.8X2*	Toxic effect of carbon monoxide from other source, intentional self-harm
T58.8X4*	Toxic effect of carbon monoxide from other source, undetermined
T58.92*	Toxic effect of carbon monoxide from unspecified source, intentional self-harm
T58.94*	Toxic effect of carbon monoxide from unspecified source, undetermined
T59.0X2*	Toxic effect of nitrogen oxides, intentional self-harm
T59.0X4*	Toxic effect of nitrogen oxides, undetermined
T59.1X2*	Toxic effect of sulfur dioxide, intentional self-harm
T59.1X4*	Toxic effect of sulfur dioxide, undetermined
T59.2X2*	Toxic effect of formaldehyde, intentional self-harm
T59.2X4*	Toxic effect of formaldehyde, undetermined
T59.3X2*	Toxic effect of lacrimogenic gas, intentional self-harm
T59.3X4*	Toxic effect of lacrimogenic gas, undetermined
T59.4X2*	Toxic effect of chlorine gas, intentional self-harm
T59.4X4*	Toxic effect of chlorine gas, undetermined
T59.5X2*	Toxic effect of fluorine gas and hydrogen fluoride, intentional self-harm
T59.5X4*	Toxic effect of fluorine gas and hydrogen fluoride, undetermined
T59.6X2*	Toxic effect of hydrogen sulfide, intentional self-harm
T59.6X4*	Toxic effect of hydrogen sulfide, undetermined
T59.7X2*	Toxic effect of carbon dioxide, intentional self-harm
T59.7X4*	Toxic effect of carbon dioxide, undetermined
T59.812*	Toxic effect of smoke, intentional self-harm
T59.814*	Toxic effect of smoke, undetermined
T59.892*	Toxic effect of other specified gases, fumes and vapors, intentional self-harm
T59.894*	Toxic effect of other specified gases, fumes and vapors, undetermined
T59.92*	Toxic effect of unspecified gases, fumes and vapors, intentional self-harm
T59.94*	Toxic effect of unspecified gases, fumes and vapors, undetermined
T60.0X2*	Toxic effect of organophosphate and carbamate insecticides, intentional self-harm
T60.0X4*	Toxic effect of organophosphate and carbamate insecticides, undetermined
T60.1X2*	Toxic effect of halogenated insecticides, intentional self-harm

T60.1X4*	Toxic effect of halogenated insecticides, undetermined
T60.2X2*	Toxic effect of other insecticides, intentional self-harm
T60.2X4*	Toxic effect of other insecticides, undetermined
T60.3X2*	Toxic effect of herbicides and fungicides, intentional self-harm
T60.3X4*	Toxic effect of herbicides and fungicides, undetermined
T60.4X2*	Toxic effect of rodenticides, intentional self-harm
T60.4X4*	Toxic effect of rodenticides, undetermined
T60.8X2*	Toxic effect of other pesticides, intentional self-harm
T60.8X4*	Toxic effect of other pesticides, undetermined
T60.92*	Toxic effect of unspecified pesticide, intentional self-harm
T60.94*	Toxic effect of unspecified pesticide, undetermined
T61.02*	Ciguatera fish poisoning, intentional self-harm
T61.04*	Ciguatera fish poisoning, undetermined
T61.12*	Scombroid fish poisoning, intentional self-harm
T61.14*	Scombroid fish poisoning, undetermined
T61.772*	Other fish poisoning, intentional self-harm
T61.774*	Other fish poisoning, undetermined
T61.782*	Other shellfish poisoning, intentional self-harm
T61.784*	Other shellfish poisoning, undetermined
T61.8X2*	Toxic effect of other seafood, intentional self-harm
T61.8X4*	Toxic effect of other seafood, undetermined
T61.92*	Toxic effect of unspecified seafood, intentional self-harm
T61.94*	Toxic effect of unspecified seafood, undetermined
T62.0X2*	Toxic effect of ingested mushrooms, intentional self-harm
T62.0X4*	Toxic effect of ingested mushrooms, undetermined
T62.1X2*	Toxic effect of ingested berries, intentional self-harm
T62.1X4*	Toxic effect of ingested berries, undetermined
T62.2X2*	Toxic effect of other ingested (parts of) plant(s), intentional self-harm
T62.2X4*	Toxic effect of other ingested (parts of) plant(s), undetermined
T62.8X2*	Toxic effect of other specified noxious substances eaten as food, intentional self-harm
T62.8X4*	Toxic effect of other specified noxious substances eaten as food, undetermined
T62.92*	Toxic effect of unspecified noxious substance eaten as food, intentional self-harm
T62.94*	Toxic effect of unspecified noxious substance eaten as food, undetermined
T63.002*	Toxic effect of unspecified snake venom, intentional self-harm
T63.004*	Toxic effect of unspecified snake venom, undetermined
T63.012*	Toxic effect of rattlesnake venom, intentional self-harm
T63.014*	Toxic effect of rattlesnake venom, undetermined
T63.022*	Toxic effect of coral snake venom, intentional self-harm
T63.024*	Toxic effect of coral snake venom, undetermined
T63.032*	Toxic effect of taipan venom, intentional self-harm
T63.034*	Toxic effect of taipan venom, undetermined
T63.042*	Toxic effect of cobra venom, intentional self-harm
T63.044*	Toxic effect of cobra venom, undetermined
T63.062*	Toxic effect of venom of other North and South American snake, intentional self-harm
T63.064*	Toxic effect of venom of other North and South American snake, undetermined
T63.072*	Toxic effect of venom of other Australian snake, intentional self-harm
T63.074*	Toxic effect of venom of other Australian snake, undetermined
T63.082*	Toxic effect of venom of other African and Asian snake, intentional self-harm
T63.084*	Toxic effect of venom of other African and Asian snake, undetermined
T63.092*	Toxic effect of venom of other snake, intentional self-harm
T63.094*	Toxic effect of venom of other snake, undetermined
T63.112*	Toxic effect of venom of gila monster, intentional self-harm
T63.114*	Toxic effect of venom of gila monster, undetermined

T63.122*	Toxic effect of venom of other venomous lizard, intentional self-harm
T63.124*	Toxic effect of venom of other venomous lizard, undetermined
T63.192*	Toxic effect of venom of other reptiles, intentional self-harm
T63.194*	Toxic effect of venom of other reptiles, undetermined
T63.2X2*	Toxic effect of venom of scorpion, intentional self-harm
T63.2X4*	Toxic effect of venom of scorpion, undetermined
T63.302*	Toxic effect of unspecified spider venom, intentional self-harm
T63.304*	Toxic effect of unspecified spider venom, undetermined
T63.312*	Toxic effect of venom of black widow spider, intentional self-harm
T63.314*	Toxic effect of venom of black widow spider, undetermined
T63.322*	Toxic effect of venom of tarantula, intentional self-harm
T63.324*	Toxic effect of venom of tarantula, undetermined
T63.332*	Toxic effect of venom of brown recluse spider, intentional self-harm
T63.334*	Toxic effect of venom of brown recluse spider, undetermined
T63.392*	Toxic effect of venom of other spider, intentional self-harm
T63.394*	Toxic effect of venom of other spider, undetermined
T63.412*	Toxic effect of venom of centipedes and venomous millipedes, intentional self-harm
T63.414*	Toxic effect of venom of centipedes and venomous millipedes, undetermined
T63.422*	Toxic effect of venom of ants, intentional self-harm
T63.424*	Toxic effect of venom of ants, undetermined
T63.432*	Toxic effect of venom of caterpillars, intentional self-harm
T63.434*	Toxic effect of venom of caterpillars, undetermined
T63.442*	Toxic effect of venom of bees, intentional self-harm
T63.444*	Toxic effect of venom of bees, undetermined
T63.452*	Toxic effect of venom of hornets, intentional self-harm
T63.454*	Toxic effect of venom of hornets, undetermined
T63.462*	Toxic effect of venom of wasps, intentional self-harm
T63.464*	Toxic effect of venom of wasps, undetermined
T63.482*	Toxic effect of venom of other arthropod, intentional self-harm
T63.484*	Toxic effect of venom of other arthropod, undetermined
T63.512*	Toxic effect of contact with stingray, intentional self-harm
T63.514*	Toxic effect of contact with stingray, undetermined
T63.592*	Toxic effect of contact with other venomous fish, intentional self-harm
T63.594*	Toxic effect of contact with other venomous fish, undetermined
T63.612*	Toxic effect of contact with Portugese Man-o-war, intentional self-harm
T63.614*	Toxic effect of contact with Portugese Man-o-war, undetermined
T63.622*	Toxic effect of contact with other jellyfish, intentional self-harm
T63.624*	Toxic effect of contact with other jellyfish, undetermined
T63.632*	Toxic effect of contact with sea anemone, intentional self-harm
T63.634*	Toxic effect of contact with sea anemone, undetermined
T63.692*	Toxic effect of contact with other venomous marine animals, intentional self-harm
T63.694*	Toxic effect of contact with other venomous marine animals, undetermined
T63.712*	Toxic effect of contact with venomous marine plant, intentional self-harm
T63.714*	Toxic effect of contact with venomous marine plant, undetermined
T63.792*	Toxic effect of contact with other venomous plant, intentional self-harm
T63.794*	Toxic effect of contact with other venomous plant, undetermined
T63.812*	Toxic effect of contact with venomous frog, intentional self-harm
T63.814*	Toxic effect of contact with venomous frog, undetermined
T63.822*	Toxic effect of contact with venomous toad, intentional self-harm
T63.824*	Toxic effect of contact with venomous toad, undetermined
T63.832*	Toxic effect of contact with other venomous amphibian, intentional self-harm
T63.834*	Toxic effect of contact with other venomous amphibian, undetermined
T63.892*	Toxic effect of contact with other venomous animals, intentional self-harm

T63.894*	Toxic effect of contact with other venomous animals, undetermined
T63.92*	Toxic effect of contact with unspecified venomous animal, intentional self-harm
T63.94*	Toxic effect of contact with unspecified venomous animal, undetermined
T64.02*	Toxic effect of aflatoxin, intentional self-harm
T64.04*	Toxic effect of aflatoxin, undetermined
T64.82*	Toxic effect of other mycotoxin food contaminants, intentional self-harm
T64.84*	Toxic effect of other mycotoxin food contaminants, undetermined
T65.0X2*	Toxic effect of cyanides, intentional self-harm
T65.0X4*	Toxic effect of cyanides, undetermined
T65.1X2*	Toxic effect of strychnine and its salts, intentional self-harm
T65.1X4*	Toxic effect of strychnine and its salts, undetermined
T65.212*	Toxic effect of chewing tobacco, intentional self-harm
T65.214*	Toxic effect of chewing tobacco, undetermined
T65.222*	Toxic effect of tobacco cigarettes, intentional self-harm
T65.224*	Toxic effect of tobacco cigarettes, undetermined
T65.292*	Toxic effect of other tobacco and nicotine, intentional self-harm
T65.294*	Toxic effect of other tobacco and nicotine, undetermined
T65.3X2*	Toxic effect of nitroderivatives and aminoderivatives of benzene and its homologues, intentional self-harm
T65.3X4*	Toxic effect of nitroderivatives and aminoderivatives of benzene and its homologues, undetermined
T65.4X2*	Toxic effect of carbon disulfide, intentional self-harm
T65.4X4*	Toxic effect of carbon disulfide, undetermined
T65.5X2*	Toxic effect of nitroglycerin and other nitric acids and esters, intentional self-harm
T65.5X4*	Toxic effect of nitroglycerin and other nitric acids and esters, undetermined
T65.6X2*	Toxic effect of paints and dyes, not elsewhere classified, intentional self-harm
T65.6X4*	Toxic effect of paints and dyes, not elsewhere classified, undetermined
T65.812*	Toxic effect of latex, intentional self-harm
T65.814*	Toxic effect of latex, undetermined
T65.822*	Toxic effect of harmful algae and algae toxins, intentional self-harm
T65.824*	Toxic effect of harmful algae and algae toxins, undetermined
T65.832*	Toxic effect of fiberglass, intentional self-harm
T65.834*	Toxic effect of fiberglass, undetermined
T65.892*	Toxic effect of other specified substances, intentional self-harm
T65.894*	Toxic effect of other specified substances, undetermined
T65.92*	Toxic effect of unspecified substance, intentional self-harm
T65.94*	Toxic effect of unspecified substance, undetermined
T71.112*	Asphyxiation due to smothering under pillow, intentional self-harm
T71.114*	Asphyxiation due to smothering under pillow, undetermined
T71.122*	Asphyxiation due to plastic bag, intentional self-harm
T71.124*	Asphyxiation due to plastic bag, undetermined
T71.132*	Asphyxiation due to being trapped in bed linens, intentional self-harm
T71.134*	Asphyxiation due to being trapped in bed linens, undetermined
T71.144*	Asphyxiation due to smothering under another person's body (in bed), undetermined
T71.152*	Asphyxiation due to smothering in furniture, intentional self-harm
T71.154*	Asphyxiation due to smothering in furniture, undetermined
T71.162*	Asphyxiation due to hanging, intentional self-harm
T71.164*	Asphyxiation due to hanging, undetermined
T71.192*	Asphyxiation due to mechanical threat to breathing due to other causes, intentional self-harm
T71.194*	Asphyxiation due to mechanical threat to breathing due to other causes, undetermined
T71.222*	Asphyxiation due to being trapped in a car trunk, intentional self-harm
T71.224*	Asphyxiation due to being trapped in a car trunk, undetermined
T71.232*	Asphyxiation due to being trapped in a (discarded) refrigerator, intentional self-harm
T71.234*	Asphyxiation due to being trapped in a (discarded) refrigerator, undetermined

X71*	Intentional self-harm by drowning and submersion
X72*	Intentional self-harm by handgun discharge
X73*	Intentional self-harm by rifle, shotgun and larger firearm discharge
X74*	Intentional self-harm by other and unspecified firearm and gun discharge
X75*	Intentional self-harm by explosive material
X76*	Intentional self-harm by smoke, fire and flames
X77*	Intentional self-harm by steam, hot vapors and hot objects
X78*	Intentional self-harm by sharp object
X79*	Intentional self-harm by blunt object
X80*	Intentional self-harm by jumping from a high place
X81*	Intentional self-harm by jumping or lying in front of moving object
X82*	Intentional self-harm by crashing of motor vehicle
X83*	Intentional self-harm by other specified means
Y21*	Drowning and submersion, undetermined intent
Y22*	Handgun discharge, undetermined intent
Y23*	Rifle, shotgun and larger firearm discharge, undetermined intent
Y24*	Other and unspecified firearm discharge, undetermined intent
Y25*	Contact with explosive material, undetermined intent
Y26*	Exposure to smoke, fire and flames, undetermined intent
Y27*	Contact with steam, hot vapors and hot objects, undetermined intent
Y28*	Contact with sharp object, undetermined intent
Y29*	Contact with blunt object, undetermined intent
Y30*	Falling, jumping or pushed from a high place, undetermined intent
Y31*	Falling, lying or running before or into moving object, undetermined intent
Y32*	Crashing of motor vehicle, undetermined intent
Y33*	Other specified events, undetermined intent
Z91.5	Personal history of self-harm

Table S5 Top 50 keywords generated by word2vec as semantically similar to 'suicide' and 'suicidal'.

	suicide+ suicidal		suicide		suicidal	
	context size = 5	context size = 15	context size = 5	context size = 15	context size = 5	context size = 15
1	suicide	suicide	self-harm	manic	ideation	ideation
2	suicidal	suicidal	suicidal	ideation	homicidal	homicidal
3	ideation	ideation	paranoid	suicidal	ideations	ideations
4	homicidal	homicidal	homicide	self-harm	paranoia	suicidality
5	self-harm	ideations	ideation	suicided	suidical	paranoia
6	ideations	manic	suicide/homicide	mania	suicidality	suidical
7	paranoia	self-harm	self-mutilation	homicidal	self-harm	delusional
8	paranoid	mania	paranoia	s/h	delusional	self-harm
9	suidical	suicidality	self-harm--plan	self-mutilation	paranoid	thoughts
10	suicidality	paranoia	manic	ptsd	suicidial	mania
11	manic	suidical	mutilation	paranoid	homocidal	manic
12	self-mutilation	self-mutilation	schizophrenic	mutilation	si/hi	si/hi
13	delusional	hypomanic	suicided	ideations	homicidal/suicidal	hypomanic
14	homicial	delusional	codependent	threats	homicial	homocidal
15	mutilation	paranoid	w/bipolar	codependent	sucidal	self-mutilation
16	sucidal	s/h	homicidal	suidical	intent/plan	suicide
17	suicidial	homocidal	self-harm,	self-destructive	avh	overdosing
18	parasuicidal	self-destructive	homicial	paranoia	ideation/plan	homicidality
19	intent/plan	mutilation	ideations	hypomanic	thoughts	paranoid
20	si/hi	si/hi	parasuicidal	parasuicidal	si/hi/intent	grandiose
21	overdosing	parasuicidal	houghts	strife	suicide	self-destructive
22	hypomanic	overdosing	then-current	flashbacks	manic	avh
23	then-current	psychotropic	overdosing	suicidality	=+@si	grandeur
24	avh	flashbacks	none--intent	none--intent	;{deny	persecutory
25	si/hi/intent	persecutory	schizophrenia	psychotropic	urge/intent	s/h
26	homicidal/suicidal	persecution	overdoses	bpad	persecution	mutilation

27	psychiatrically	threats	suidical	dwf	self-mutilation	ruminations
28	ideation/plan	isolative	hypomanic	"manic	hypomanic	parasuicidal
29	=+@si	thoughts	ptsd	isolative	=+@suicidal	suicidial
30	mania/hypomania	manic/hypomanic	delusional	i/j	psychiatrically	manic/hypomanic
31	homocidal	grandiose	mania/hypomania	j/i	suicidal/homicidal	future-oriented
32	homicide	helplessness	rapes	homocidal	ideation{	persecution
33	self-destructive	mania/hypomania	threats	homicide	overdosing	psychotic
34	suicidal/homicidal	suicided	s/h	plan/intent	parasuicidal	"depressed
35	mania	ptsd	overdose	persecutory	grandeur	psychotropic
36	urge/intent	avh	psychiatrically	overdosing	persecutory	hopelessness
37	plan/intent	plan/intent	rape	suicidal/homicidal	plan/intent	helplessness
38	persecution	"hopeless	saam	homosexuality	grandiose	illusions
39	self-harm--plan	psychotic	sucidal	reckless	mania/hypomania	isolative
40	thoughts	homicidality	self-destructive	manic/hypomanic	then-current	mood-congruent
41	s/h	fidgetiness	"schizophrenia	mania/hypomania	manic/hypomanic	flashbacks
42	psychotropic	future-oriented	suicidality	nihilistic	mutilation	mania/hypomania
43	plans/intent	suicidal/homicidal	bpad	helplessness	delusions	obsessions
44	persecutory	"manic	intent/plan	hypervigilence	hopelessness	urge/intent
45	threats	hypervigilance	mania	fidgetiness	plans/intent	"hopeless
46	grandeur	ruminations	si/hi/intent	estrangement	preoccupations	pressured
47	suicidal/	hopelessness	murder	"hopeless	mania	fidgetiness
48	;{deny	obsessions	psychotropic	discord	ideation/intent/plan	worthless
49	overdoses	suicidial	suicide,	=--@plan	suicideal	dysphoric
50	self-harm,	hopless	avh	self-harm--plan-no-intent-no--past	ruminations	hypervigilance

Table S6 Query terms used to retrieve suicidal ideation and suicide attempt.

<table>
<tr><th>Outcome</th><th>Query terms</th></tr>
<tr><td>Suicidal Ideation</td><td>suicid(al|e) idea(tion|s)
suicid(al|e) thought(s)
thought(s) of suicide
want(s|ing) to die
(thoughts|think|want)(s|ing) (of|to) (take|end)(ing) (my|his|her) (own) life
(thoughts|think|want)(s|ing) (of|to) (kill|shoot|hang|poison)(ing) (myself|himself|herself)
feel(s|ing) (very) suicidal</td></tr>
<tr><td>Suicide attempt</td><td>suicid(al|e) attempt
(try|tried|tries|attempted|attempts) (of|to) (take|end)(ing) (my|his|her) (own) life
(try|tried|tries|attempted|attempts) (of|to) (kill|shoot|hang|poison)(ing) (myself|himself|herself)</td></tr>
</table>

Table S7 Comparative analysis for extracting the top K highest ranked suicidal ideation (SI) and suicide attempt (SA) patients using various configurations of the suicide label assignment method. The "All retrieved" experiments include all the patients retrieved by the NLP system while the "w/ 1+ positive" ones use only patients with at least one positive suicide mention in their notes. "NLP" and "NLP+ICD" experiments are associated with methods using p_{rank} and $p_{NLP+ICD}$, respectively.

		All retrieved				w/ 1+ positive			
		NLP		NLP+ICD		NLP		NLP+ICD	
		mean	sd	mean	sd	mean	sd	mean	sd
SI	Top K (P@K=90)	926.83	104.83	1,162.55	80.61	975.01	106.22	1,228.01	84.03
	Top K (P@K=80)	3,094.51	144.54	5,782.72	192.52	3,135.62	138.99	5,945.07	186.15
SA	Top K (P@K=90)	384.52	32.27	495.58	55.82	394.59	37.89	526.77	67.84
	Top K (P@K=80)	818.00	64.90	1,663.73	114.22	839.86	66.88	1,693.19	122.55

Table S8 Suicidal ideation (SI) and suicide attempt (SA) cases extracted from: 1) the top ranked patients by the NLP system, 2) patients manually labeled as cases, 3) patients with positive assertions for suicidal ideation and suicide attempt in their psychiatric forms, and 4) patients with ICD10CM codes for self-injurious thoughts and behaviors.

Resource	SI	SA
NLP, top K, P@K=90	1,209	380
NLP, top K, P@K=80	5,342	1,384
Chart review (cases)	991	718
Psychiatric forms (cases)	5,232	4,275
ICD10CM codes	20,037	15,729
